# An Efficient *Aequorea victoria* Green Fluorescent Protein for Stimulated Emission Depletion Super-Resolution Microscopy

**DOI:** 10.3390/ijms23052482

**Published:** 2022-02-24

**Authors:** Barbara Storti, Benedetta Carlotti, Grazia Chiellini, Martina Ruglioni, Tiziano Salvadori, Marco Scotto, Fausto Elisei, Alberto Diaspro, Paolo Bianchini, Ranieri Bizzarri

**Affiliations:** 1 NEST, Scuola Normale Superiore and Istituto Nanoscienze-CNR, Piazza San Silvestro 12, 56127 Pisa, Italy; ranieri.bizzarri@unipi.it; 2 Department of Chemistry, Biology and Biotechnology and CEMIN, University of Perugia, Via Elce di Sotto 8, 06123 Perugia, Italy; benedetta.carlotti@unipg.it (B.C.); fausto.elisei@unipg.it (F.E.); 3 Department of Surgical, Medical and Molecular Pathology and Critical Care Medicine, University of Pisa, Via Roma 65, 56126 Pisa, Italy; grazia.chiellini@unipi.it (G.C.); m.ruglioni1@studenti.unipi.it (M.R.); t.salvadori@studenti.unipi.it (T.S.); 4 Nanoscopy, CHT, Istituto Italiano di Tecnologia, Via E. Melen 83, 16152 Genova, Italy; marco.scotto@iit.it (M.S.); alberto.diaspro@iit.it (A.D.); paolo.bianchini@iit.it (P.B.); 5 DIFILAB, Dipartimento di Fisica, Università degli Studi di Genova, Via Dodecaneso 33, 16146 Genova, Italy

**Keywords:** stimulated emission depletion (STED), fluorescent protein, super-resolution, ultrafast spectroscopy, excited state absorption (ESA), Vimentin

## Abstract

In spite of their value as genetically encodable reporters for imaging in living systems, fluorescent proteins have been used sporadically for stimulated emission depletion (STED) super-resolution imaging, owing to their moderate photophysical resistance, which does not enable reaching resolutions as high as for synthetic dyes. By a rational approach combining steady-state and ultrafast spectroscopy with gated STED imaging in living and fixed cells, we here demonstrate that F99S/M153T/V163A GFP (c3GFP) represents an efficient genetic reporter for STED, on account of no excited state absorption at depletion wavelengths <600 nm and a long emission lifetime. This makes c3GFP a valuable alternative to more common, but less photostable, EGFP and YFP/Citrine mutants for STED imaging studies targeting the green-yellow region of the optical spectrum.

## 1. Introduction

The modern foundation of biochemistry and molecular biology owes much to the ability of far-field microscopy to reveal the functional architecture of eukaryotic cells, bacteria, and viruses. Yet, until very recently, the spatial resolution of far-field microscopy was believed to be limited by the intrinsic wave nature of light, which generates diffraction whenever light interferes with objects of size similar or smaller than its wavelength [1]. According to Abbe’s theory, light of a wavelength λ, focused by a lens of numerical aperture NA, cannot distinguish sample features any closer than distance d = λ/(2NA) on the focal plane, because diffraction merges their images into a single blur [2]. In this context, nanoscale resolution can be achieved only by very short wavelengths like in electron microscopy (λ~0.04 nm), at the price of using ionizing radiations that restrict imaging to non-vital samples and often require a complex experimental preparation. Conversely, fluorescence microscopy makes use of non-ionizing optical wavelengths that may allow for the imaging of living samples, but Abbe’s theory limits its resolution to about 180–200 nm, a scale that often does not comply with the meso-to-nanoscale size of intracellular and extracellular biological systems [3]. Yet, fluorescence microscopy is particularly suitable for applications in biology, as it enables multiplex detection of several functional parameters with an exquisite sensitivity that may reach the single molecule limit [4]. The application range of fluorescence microscopy in biology has been greatly expanded by the discovery of the Green Fluorescent Protein (GFP) and its variants (FPs), i.e., genetically encodable fluorescent probes that can be fused to almost any target protein in cellulo and which are leveraged to study the real-time dynamics of bioprocesses with unprecedented details and in their actual biological context [5].

It took about a century for establishing that the Abbe’s diffraction limit holds from a physical point of view, but it can be effectively circumvented by adding additional information when forming an image [1,6]. The 2014 Nobel prize in Chemistry awarded three scientists whose seminal work demonstrated “super-resolution” (i.e., below Abbe’s limit) imaging by leveraging new detection modalities which combine localization precision or shaping of the illumination beam with the emission bistability of fluorophores under certain conditions. Consequently, in the last few years, several important studies revealed hidden details of biological systems at nanoscale by means of super-resolution optical imaging.

Among the toolbox of super-resolution techniques, STimulated Emission Depletion (STED) combines widespread application to many fluorescent probes with the capability of in vivo imaging. Technically, STED limits the spatial region of effective excitation by means of excited state depletion by stimulated emission, which is triggered by strong laser illumination on the low-energy tail of the dye’s emission spectrum [7]. At a spatial level, depletion is obtained by a donut-shaped beam, which is superimposed upon the focused illumination spot. At a spatial level, depletion is obtained by a donut-shaped beam which is superimposed upon the focused excitation spot. Most excited molecules which encounter the depletion beam are forced back to the ground state, releasing two identical photons whose wavelength is outside the collection range. This optical arrangement implies that only the fluorescence of excited molecules at the center of the donut is revealed, as they do not undergo stimulated emission. Raster-scanning the sample allows the sequential registration of fluorescence from only those dyes that, at each step, find themselves in the center of the donut [7]. It can be demonstrated that STED resolution scales with the square root of depletion intensity I_d_ at the donut crest according to the reformulated Abbe’s equation d = [λ/(2NA)]/[1 + I_d_/I_s_]^0.5^ [8]. I_s_ is called the saturation intensity, and corresponds to the depletion intensity at which half of the excited fluorophores are shut off. Thus, the resolution d has in principle no limit, as d→0 for I_d_ ≫ I_s_. From the STED theory, I_s_ = hν/στ [9], with hν/σ denoting the energy of depletion photons divided by the stimulated emission cross-section, and τ is the lifetime of the excited state.

The simplest technical way to implement STED is by using a confocal microscope supplied with a continuous wave depletion beam (CW-STED). In such a system, the use of pulsed excitation and collecting fluorescence after a gate time τ_g_ determines a significant improvement in resolution owing to a larger spatial on-off contrast (gated-STED or g-STED) [10]. Modern confocal microscopes allow fast modulation of scanning to comply with a wide range of biological dynamics in living cells. Additionally, any fluorophore can undergo fluorescence depletion of the excited state by the stimulated emission phenomenon. Yet, the universal use of STED imaging is limited by the photophysical resistance of the fluorophore. In principle, an ideal STED fluorophore should immediately restore fluorescence once it enters into the donut minimum. This is feasible only if the depletion beam does not promote significant excited state absorption (ESA), which almost inevitably leads to long-lived dark states and/or bleaching pathways [11,12]. A second valuable property of the fluorophore is to possess a long lifetime, and a large cross-section at the depletion wavelength, in order to reduce I_s_ and the associated photophysical stress to achieve a given resolution. Nowadays, several synthetic fluorophores comply with these properties and enable high resolutions at rather low depletion powers by STED [13]. Unfortunately, delicate photophysics and rather short lifetimes prevented most FPs from STED applications [14,15], partially hampering STED nanoscopy of living samples for which the use of FPs is most valuable. 

In the green-yellow region of the spectrum, STED was mostly applied to EGFP (F64L/S65T GFP) [16,17,18], EYFP (S65G/V68L/S72A/T203Y GFP) [19], and Citrine (Q68M EYFP) [20] by using 556–592 nm depletion wavelengths. EGFP and YFP represent popular fluorophores for biological studies, but their optical properties are not fully optimized for STED. EGFP displays an intermediate lifetime of about 2.6 ns [21] and is characterized by ESA starting from 580 nm [17]. The lifetime of YFP and Citrine is longer than EGFP (3.3 ns), but the T203Y replacement is known to promote long-lived dark states [22,23] and lower photochemical stability of the chromophore [24]. In this work, we show for the first time that the green mutant c3GFP (F99S/M153T/V163A GFP) represents a valuable alternative to EGFP/YFP/Citrine for STED studies of living and fixed cellular samples, on account of its long lifetime and the absence of ESA at depletion wavelengths < 600 nm. Of note, our findings came from a rational characterization approach combining steady-state, time-resolved, and ultrafast spectroscopy with CW g-STED measurements.

## 2. Results

### 2.1. Steady-State Spectrophotometric Measurements

**Figure 1:**
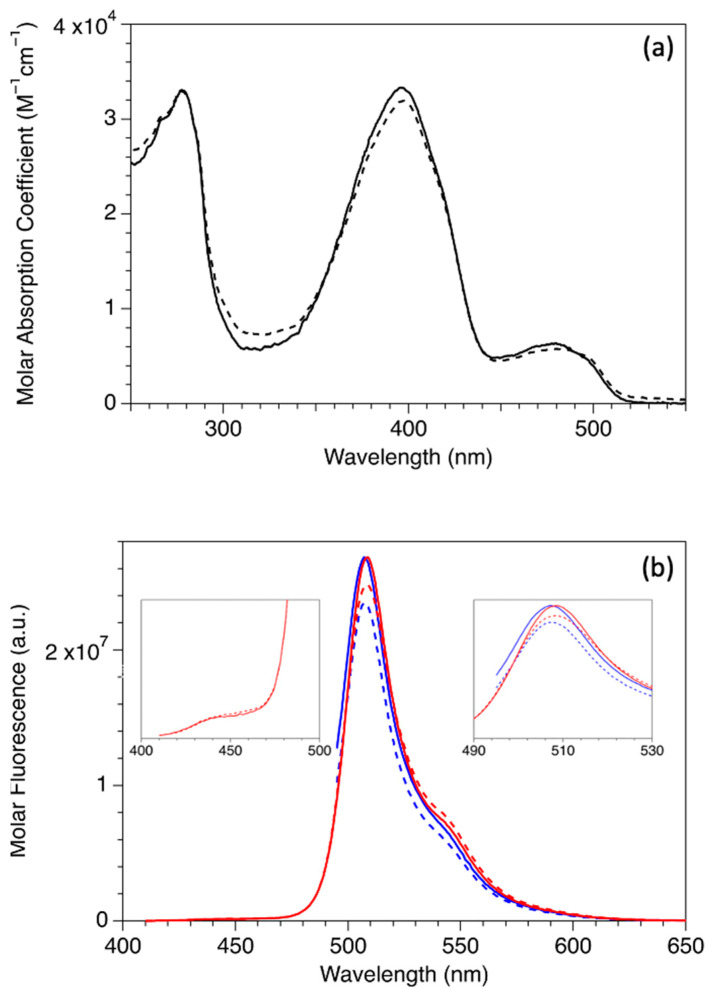
Steady-state spectroscopic properties of c3GFP. (**a**) Absorption spectra of c3GFP at pH 5.2 (full line) and pH 8.0 (dashed line). (**b**) Fluorescence emission spectra of c3GFP by exciting at 400 nm (red full line: pH 5.2; red dashed line: pH 8.0), or at 490 nm (blue full line: pH 5.2; blue dashed line: pH 8.0). Insets: expansions of the 400–500 (left) and the 490–530 nm regions to highlight the peaks’ positions.

In the 250–700 nm range, the absorption spectrum of c3GFP contains three main bands, at 278 nm, 397 nm, and 481 nm, respectively (Figure 1a). Similarly to all *Aequorea* proteins, the band at 278 nm is originated by the aromatic amino acids. We calculated ϵ(278) = 32,900 M^−1^cm^−1^, in excellent agreement with the value previously reported for EGFP (ϵ(278) = 32,650 M^−1^cm^−1^ [25]), which contains the same aromatic amino acids of c3GFP. The two bands in the visible region pertain to the protonated (397 nm, ϵ = 31,900 M^−1^cm^−1^) and deprotonated chromophore (479 nm, ϵ = 5800 M^−1^cm^−1^). Remarkably, neither the intensity nor the shape of the two absorption bands almost changed between pH 5.2 and pH 8. Analogously, excitation near the absorption maxima led to nearly pH-independent fluorescence emission (Figure 1b).

The same pH-independent behavior of absorption and fluorescence is shown by the ancestor protein wtGFP and is partially shared by some T203 mutants [25,26]. This picture can be interpreted in light of the 2S model of chromophore protonation [25]. According to the 2S model, c3GFP is characterized by a strong thermodynamic coupling between the proton exchange of chromophore and that of the nearby residue Glu^222^. Ionization of Glu^222^ occurs at a rather low pH and the resulting anionic Glu^222^ “buffers” the chromophore into an almost pH-independent equilibrium between protonated (A) and deprotonated (B) chromophore states. 

**Scheme 1:**
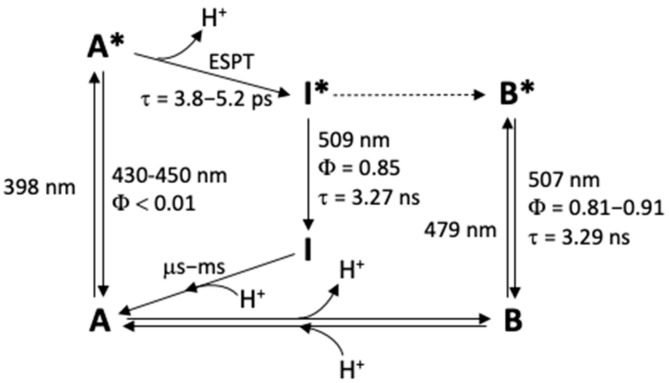
Photophysical processes of c3GFP chromophore. The role of the Excited State Proton Transfer in determining the protein fluorescence from the neutral state A is highlighted. Spectral, quantum yield, and lifetime values were determined in this work. Solid arrows: active processes. Dashed arrows: infrequent or non-active processes.

Of note, the emission bands of **A** and **B** showed close but distinguishable maxima at 509 and 507 nm, respectively (Figure 1b, right inset). This difference likely witnesses a photocycle analogous to wtGFP (Scheme 1) [27]. Excitation of **B** leads to direct emission from B* excited state. Excitation of the neutral chromophore in **A** activates two emission channels (Scheme 1). The direct emission from A* may occur around 440–450 nm, but it has a very low efficiency (Figure 1b, left inset). A much more effective channel stems from the increased acidity of the phenol group in A* [28], which triggers an Excited State Proton Transfer (ESPT) from A* to a nearby acceptor (presumably anionic Glu^222^ like in wtGFP). In such a way, A* evolves to I*, an intermediate excited state where the chromophore is anionic like in B* but its surrounding residues are in the relaxed form typical of **A** owing to the short timescale of I* decay (a few ns) that does not allow for the rearrangement of the chromophore environment driven by phenol deprotonation (e.g., flipping of the lateral chain of Thr^203^) [29]. This explains why I* emits at wavelengths similar, but not equal, to B*. Eventually, the ground state **I** quickly evolves to much more stable **A** [26] in ~ms time scale [30] (Scheme 1).

Interestingly, the emission quantum yields (Φ) by exciting **B** (pH 5.2: Φ = 0.91; pH 8.0: Φ = 0.81) nearly paralleled those found by exciting **A** (pH 5.2: Φ = 0.85; pH 8.0: Φ = 0.85), suggesting that the ESPT process is very efficient, as well as the I* state is electronically close to the B* state. The same phenotype has been observed for wtGFP, although its quantum yields are somewhat lower (**A**: Φ = 0.78 [31]; pH 8.0: Φ = 0.79 [32]).

### 2.2. Time-Resolved Spectrophotometric Measurements

**Figure 2:**
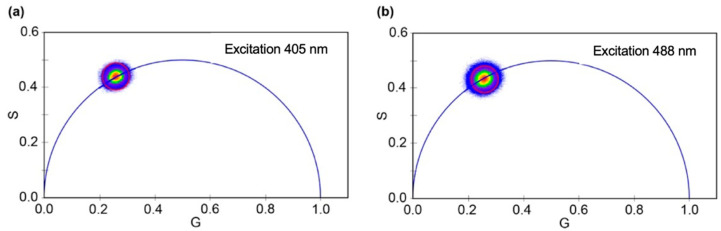
Frequency domain analysis of c3GFP fluorescence lifetime (for theory: see [33]). Phasor plots of c3GFP at 80 MHz modulation excitation of **A** (λ = 405 nm, panel (**a**)) and **B** (λ = 488 nm, panel (**b**)). G and S are the cosine and sine components of the Fourier Transforms of the lifetime decay calculated at 80 MHz. Each dot (phasor) corresponds to the decay of a single pixel in the original acquisitions. For both 405 and 488 nm panels, the phasor clouds almost lay on the same position of the universal circle, witnessing similar monoexponential decays (**A:** τ = 3.27 ± 0.02 ns, **B**: τ = 3.29 ± 0.02 ns).

The >100 ps fluorescence decays of c3GFP were determined by frequency domain spectrofluorometry upon illumination at λ = 405 and λ = 488 nm, in order to excite the **A** and **B** state, respectively. In both cases, the phasor plot analysis showed that phasor clouds of the modulated fluorescence emission laid on the universal phasor circle (Figure 2), indicating a monoexponential decay from the excited state [33]. Remarkably, **A** and **B** are characterized by nearly coincident emission lifetimes (**A**: τ = 3.27 ± 0.02 ns, **B**: τ = 3.29 ± 0.02 ns), consistently with the expected structural similarity of their actual emitting states (Scheme 1). Of note, EGFP is characterized by an appreciably shorter decay by excitation at 488 nm (2.6 ns [21]), which targets its unique emitting state **B**.

**Figure 3:**
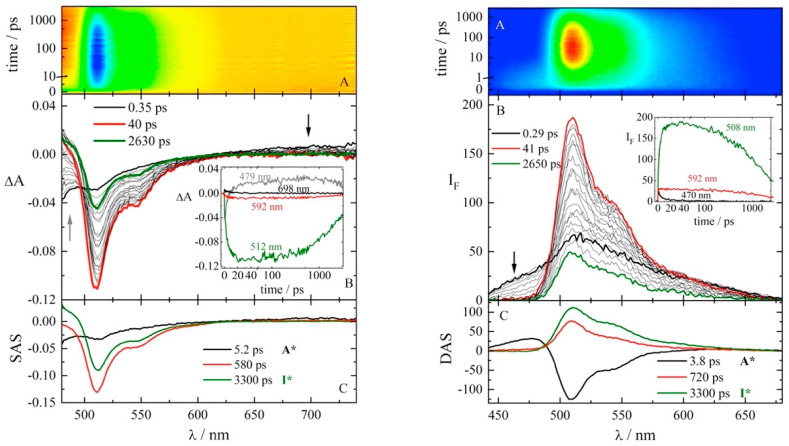
Ultrafast spectroscopy of c3GFP.Femtosecond transient absorption (**left**) and fluorescence up conversion (**right**) measurements of c3GFP at pH 8.0 with λ_pump_= 400 nm. Panel A: experimental 3D matrix of the ΔA/I_F_ signal as a function of wavelength and time; Panel B: lines are the representative spectra at different delay times as detailed by the legend, with the arrows indicating the temporal variation of the ΔA/I_F_ signal, (in the main graph) and representative kinetics at different wavelengths (in the inset); Panel C: lines represent the Decay Associated Spectra (DAS) obtained by Global Analysis and Species Associated Spectra (SAS) obtained by Target Analysis, with the lifetimes of the components revealed by the fitting detailed in the legend.

The excited state dynamics of c3GFP on a shorter time window as investigated by means of ultrafast spectroscopy, performing experiments both in absorption and in emission. Figure 3 shows the femtosecond transient absorption (TA) as well as the broadband fluorescence up conversion (FUC) results obtained at pH 8 by employing a 400 nm pump laser. These measurements were very informative, allowing the entire time-resolved spectra at different delays after photoexcitation to be obtained. In the graphs in Figure 3, the time-resolved spectra and the kinetics are reported in panel B (and relative inset) together with the entire data matrix as differential absorbance/fluorescence intensity vs. wavelength and time in panel A. The lifetimes and the spectra of the exponential components revealed through global fitting are shown in panel C. The TA spectra (Figure 3, panel B on the left) were dominated by negative ΔA signals due to stimulated emission. Positive ESA was observed only below 500 nm in the spectra recorded at long delays after excitation and above 650 nm at early delays with very low intensity. No ESA was revealed in the region between 540 and 600 nm, and particularly at 592 nm, where CW-STED irradiation is generally performed.

Of note, both the transient absorption and the time-resolved fluorescence spectra showed a significant evolution right after photoexcitation (Figure 3). In particular, the 450–500 nm emission at short times quickly decayed, while an ultrafast (~ps) rise of the 510 nm emission was observed. On account of the selective excitation of **A** at 400 nm, this ultrafast dynamic should be associated with the ESPT through which the anionic I* species is produced from the neutral A* form [27]. The I* fluorescence at 510 nm subsequently decayed in the nanosecond time scale.

To get a quantitative insight of the excited state dynamics, we carried out global fitting of both the TA and FUC. Data were adequately represented by three exponential components. Free fitting returned a lifetime of about 3 ns for the slowest component, similar to the value obtained by frequency domain fluorometry (Figure 2). Yet, both TA and FUC were characterized by a time window of 2.9 ns, which prevented an accurate identification of this lifetime from experimental data. Accordingly, we carried out a further fit iteration by fixing the slowest component to the measured lifetime of c3GFP, 3.3 ns. Under this condition, the intermediate component showed a 580 ps (TA)–720 ps lifetime, whereas the fastest component was characterized by a lifetime of few ps (5.2 ps from the TA and 3.8 ps from the FUC). The latter figure is in excellent agreement with the reported ESPT time from the neutral excited chromophore [34]. The Decay Associated Spectra (DAS) obtained through Global Analysis of the FUC confirmed the identification of the fast component with the ESPT from the A* state, given the positive band under 480 nm that should be attributed to direct emission from A*. Additionally, the negative profile of DAS of the fast component at >480 nm, compared to the positive and specular DAS profiles of the other two components in the same spectral region, suggested that ESPT was the precursor of both the intermediate and slow-decaying forms. For this reason, the TA data were analyzed through Target Analysis by introducing a branching after the first transient, and thus, obtaining the relative Species Associated Spectra (SAS). Of note, both DAS and SAS of intermediate and slow components displayed almost identical profiles, being different only for relative intensities. This feature suggested the identification of both components with I*, albeit in different environmental conditions. Neat I* corresponds to the slow component, whereas the intermediate form refers to a self-quenched I* state, which likely stems from the high concentration (100 μM) of c3GFP3 in the solution for ultrafast spectroscopy. Indeed, the parent GFP protein was found to dimerize significantly above 60 μM concentration [26] and a similar behavior is expected also for the close variant c3GFP. It was recently demonstrated that EGFP (which shares similar emission properties with c3GFP) is 5–10-fold self-quenched at high concentrations compared to the dilute solution [35,36]. This order of self-quenching is consistent with the observed shortening of lifetime from 3.3 ns (slow component) to 600–700 ps (intermediate component). The self-quenching hypothesis rationalizes the presence of a neat monoexponential decay of fluorescence in dilute solution, as witnessed by the almost perfect coincidence of the phasor cloud with the universal circle in the phasor plot (Figure 2). Under this hypothesis, the photophysical behavior of c3GFP is fully recapitulated by Scheme 1. 

### 2.3. In Vitro Depletion Properties of c3GFP and Comparison with EGFP

**Figure 4:**
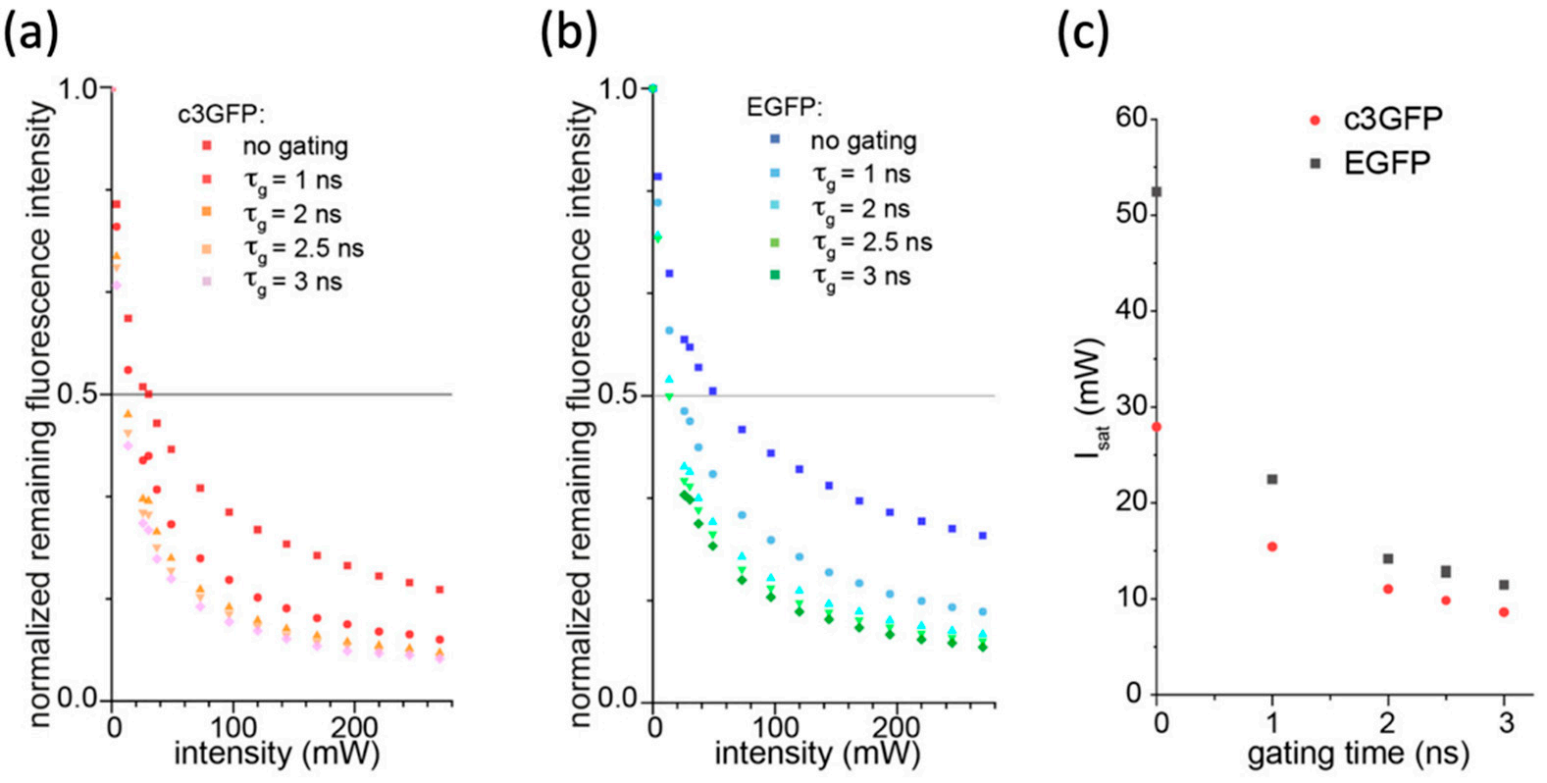
Depletion analysis of c3GFP and EGFP in solution. (**a**) Depletion curve of c3GFP at different gating conditions. (**b**) Depletion curve of EGFP at different gating conditions. (**c**) Dependence of the saturation intensity from the gating time.

Prior to experiments in cells, we set out to determine the most appropriate STED conditions for cGFP3 and compare them with the EGFP benchmark. Accordingly, we carried out depletion analysis on both proteins in solution by superimposing the excitation and depletion beams (both of Gaussian shape) in the STED microscope and hereupon collecting the fluorescence at different depletion intensities (Figure 4) [37,38]. The excitation wavelength was set to 488 nm, while STED is at 592 nm, and we explored variable time gating (τ_g_) of collected emission collection from no gating (τ_g_ = 0) to τ_g_ = 3 ns, the latter close to the lifetime of c3GFP.

As expected, the collected emission always decreased upon increasing the depletion power (Figure 4a). Without gating, the depletion power that reduces the original fluorescence by 50% corresponds to the actual I_sat_ of the fluorophore. c3GFP was characterized by a significantly lower I_sat_ than EGFP (EGFP: I_sat_ = 52 mW, c3GFP: I_sat_ = 28 mW Figure 4). This difference could be at least partially explained by the longer excited-state lifetime of c3GFP. By using the square root law of resolution (see Introduction), at common depletion powers of 180–200 mW, these I_sat_ values reflect 2.1–2.2× and 2.5–2.7× resolution gains for EGFP and c3GFP, respectively, compared to the diffraction limit.

At τ_g_ > 0, less depletion power was required to scale the original fluorescence by 50% (Figure 4b), in keeping with the results of Appendix A, where we show a simple theoretical analysis (Appendix A). The threshold value I_sat_(τ_g_) measures the intensity required to achieve the on/off contrast between the donut minimum and maximum in STED, and determines the ultimate resolution [10]. This consideration would prompt for adopting large τ_g_, to reduce I_sat_ and—consequently—to obtain a given resolution by a less intense depletion beam. This, in turn, would alleviate the photophysical/chemical stress of the protein fluorophore given by the depletion beam. Yet, increasing τ_g_ leads to a minor collected emission from undepleted molecules, which in turn decreases the S/R ratio and, ultimately, degrades the resolution [39]. In Appendix A, we show that the gated emission scales with exp(−τ_g_/τ) with respect to the original fluorescence. This means that at τ_g_ = 1 ns the collected fluorescence of c3GFP and EGFP are reduced to 74% and 68% of the non-gated values, respectively. These figures scale to 55% and 46% for τ_g_ = 2 ns, and to 41% and 32% for τ_g_ = 3 ns. In the next section, we show that the STED resolution of c3GFP increased from τ_g_ = 1 ns to τ_g_ = 2 ns, but this trend was reversed for τ_g_ = 3 ns on account of the higher noise in the STED image. Thus, τ_g_ = 2 ns was adopted for c3GFP as a practical compromise between gate time and S/N. 

### 2.4. In Cellulo STED Imaging of c3GFP and Comparison with EGFP

**Figure 5:**
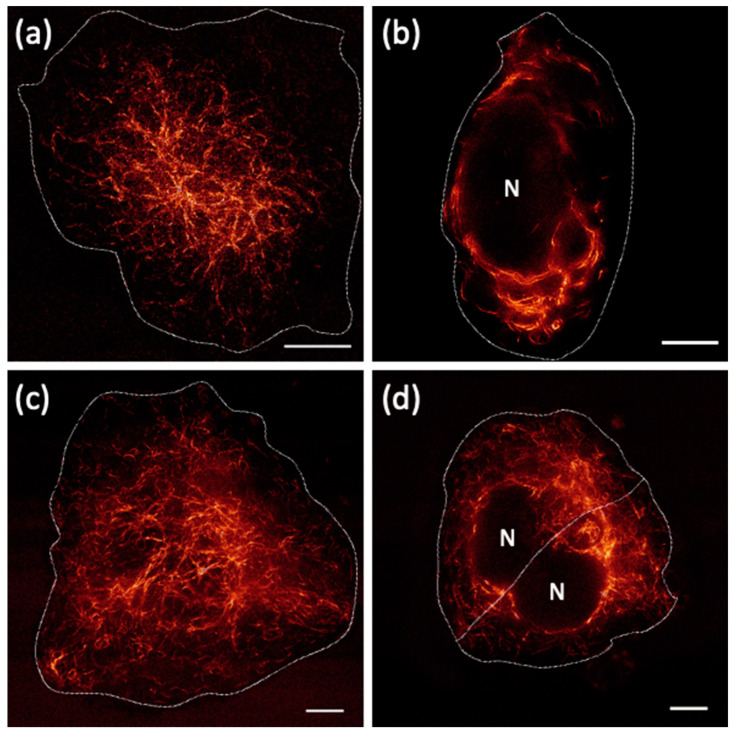
Cellular distribution of Vim-c3GFP. STED images of Vim-c3GFP (**a**,**b**) and Vim-EGFP (**c**,**d**) collected at the basal (**a**,**c**) and mid-cell (**b**,**d**) planes to show the vimentin IF arrangement in the cytoplasm (immediately above the basal membrane) and around the nucleus, respectively. The cell perimeter is highlighted by a white dotted line and the position of the nucleus is marked by N. Scale bars: 5 μM.

In order to check the capability of c3GFP to perform as a probe for STED imaging in biological samples, we engineered a fusion construct of c3GFP and Vimentin (Vim-c3GFP). Vimentin is an intermediate filament (IF) protein, which is recognized as the main cytoskeletal component responsible for maintaining cell integrity by embedding and shielding organelles and enabling the cell’s mechanical flexibility [40]. Vimentin also plays a relevant role in the mechanotransduction of signals from the exterior of the cell to its core [41], as well as in the epithelial-to-mesenchymal transition (EMT) reprogramming, which is associated with the acquisition of a migratory and invasive tumor cell phenotype [42]. By a complex multistep process, Vimentin assembles into IFs of about 10 nm diameter [43], which can also stick together to yield thicker IF bundles. Owing to their relevance in mechanobiology and tumor progression, Vimentin IFs have become a popular target of super-resolution imaging [10,44]. In our experiments, we expressed Vim-c3GFP in two Non-Small Cell Lung Cancer (NSCLC) cell lines, namely H1975 and A549, which represent classical epithelial cell models for EGFR-mutated and K-Ras-mutated NSCLC adenocarcinoma, respectively. Upon transient transfection in cells, Vim-cGFP3 afforded strongly fluorescent IFs throughout the cytoplasm and near the nucleus (Figure 5a,b). Fixation procedure by paraformaldheyde (PFA 2%) did not lead to appreciable fluorescence quenching (not shown). 

**Figure 6:**
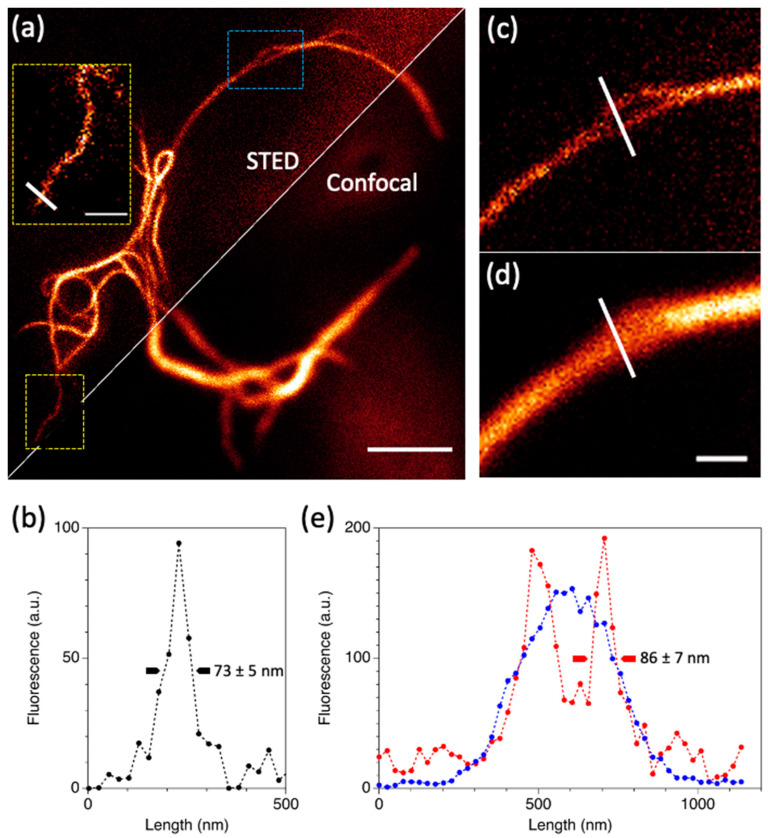
STED performances of c3GFP. (**a**) STED and confocal images of Vim-c3GFP in a H1975 cell (scale bar: 5 μM); the inset shows a zoom of the IF enclosed in the yellow dotted square region of the main panel (scale bar: 1 μM). (**b**) Fluorescence profile of the Vim-c3GFP IF along the white line visible in the inset of panel (**a**): the FWHM obtained by Gaussian fitting is reported. (**c**) STED and (**d**) confocal zooms of the cyan dotted square region of panel (**a**); scale bars: 1 μM. (**e**) Fluorescence profiles of the Vim-c3GFP IF along the white lines visible in (**c**,**d**) panels; STED: red line/markers; confocal: blue line/markers; the FWHM obtained by Gaussian fitting of the second STED peak is reported.

On account of the depletion data in vitro, we carried out g-STED by imposing τ_g_ = 2 ns and by using 190 mW of 592-nm depletion light. This led to observing IFs as thin as ~70 nm (Figure 6a,b), in excellent agreement with previous g-STED results making use of synthetic dyes [10]. Under the conservative assumption of imaging a single 10 nm-diameter IF, our finding would return a resolution of ~70 nm for g-STED applied to c3GFP3. g-STED imaging afforded a strong (4–4.5×) resolution enhancement over confocal imaging, enabling detection of subtle IF features which were otherwise hidden in confocal images (Figure 5c,d). Remarkably, this figure is in excellent agreement with the expected gain in resolution (4.1×) for I_sat_ (τ_g_ = 2 ns) = 11 mW by assuming the square root resolution law (see Introduction).

**Figure 7:**
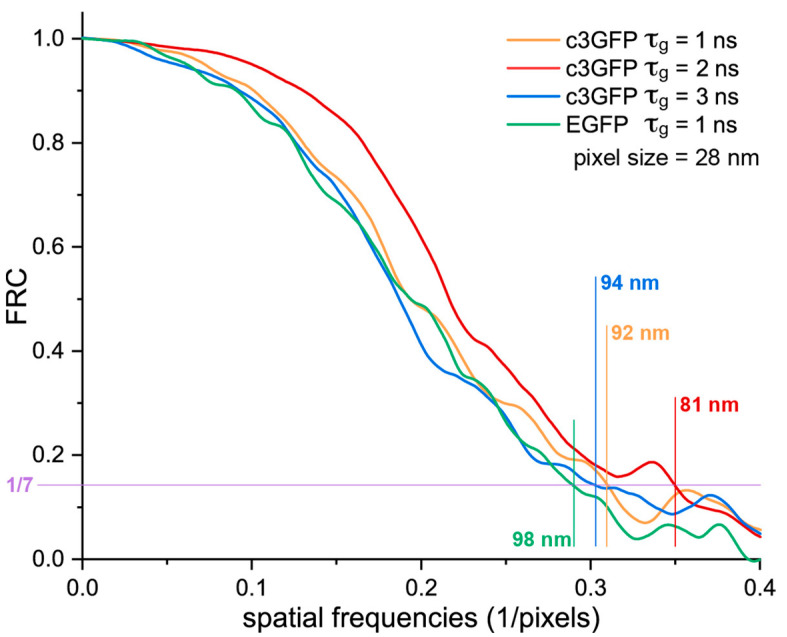
Fourier Ring Correlation Analysis of g-STED images. FRC curves relevant to Vim-c3GFP and Vim-EGFP are reported for different gate times. The intercept of each FRC curve with the standard threshold 1/7 identifies the cutoff frequency for that acquisition and, in turn, the image resolution (For theory, see:[39]).

To get a deeper insight on the effect of gate time on resolution, we carried out Fourier Ring Correlation (FRC) analysis on a set of STED images acquired at I_d_ = 190 mW and τ_g_ = 1, 2, and 3 ns (Figure 7). FRC allows to determine an estimate of the absolute resolution value by measuring the degree of correlation of two images at different spatial frequencies [39]. The correlation curve starts from unity at low spatial frequencies, for which very high correlation is observed between the two images. At spatial frequencies higher than the effective frequency which identifies the image resolution (*cutoff frequency*), non-correlated noise realizations dominate and the curve levels off to zero. The cutoff frequency is identified by a threshold in the correlation, which is usually set to 1/7 [39]. As expected, we found out a significant increase in resolution between τ_g_ = 1 ns and τ_g_ = 2 ns, as it changed from 92 nm to 81 nm (Figure 7). A further increase in gate time (τ_g_ = 3 ns) reversed this trend (94 nm resolution). This is attributable to the decrease of collected fluorescence. The higher noise in the image overcompensates the minor gain in the I_d_/I_s_ ratio and degrades the effective resolution of the image [39]. Of note, the FRC resolution at τ_g_ = 2 ns is in good agreement with the thinnest IFs detected in STED images. Yet, FRC affords a more reliable estimate of STED resolution because it is collected over a whole image instead of on a single localized feature. 

**Figure 8:**
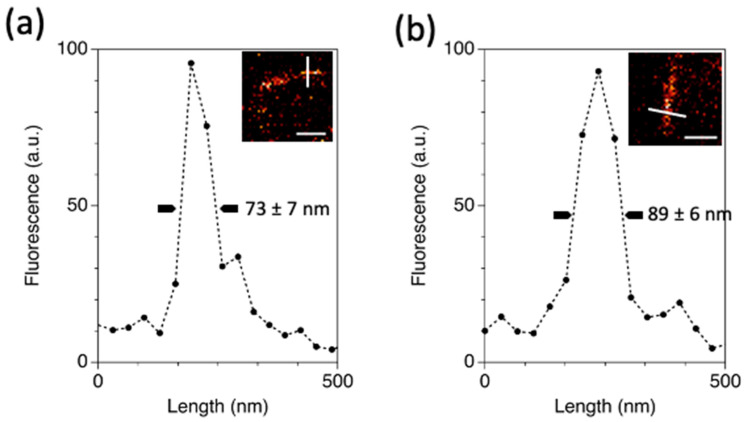
Comparison between c3GFP and EGFP under STED imaging conditions. (**a**) Fluorescence profile of a representative Vim-c3GFP IF along the white line visible in the inset (scale bar: 500 nm): the FWHM obtained by Gaussian fitting is reported. (**b**) Same as for (**a**) but Vim-c3GFP is replaced by Vim-EGFP.

In order to compare c3GFP with EGFP, we also engineered Vimentin fused to EGFP (Vim-EGFP). Transient transfection of Vim-EGFP gave a phenotype of fluorescent IFs similar to Vim-c3GFP (Figure 5c,d), confirming the validity of our benchmark construct. Next, g-STED imaging of both proteins was carried out by adopting a more favorable S/N condition for the faster-decaying EGFP, i.e., τ_g_ = 1 ns, for which this protein retains still >50% of the initial fluorescence. In this circumstance, the minimum detected size of IFs was ~90 nm for Vim-EGFP (Figure 8a) and ~70–80 nm for Vim-c3GFP (Figure 8b). FRC confirmed the lower resolution obtained for Vim-EGFP compared to Vim-c3GFP, although the difference between the two proteins was less marked (Figure 7). Consistently with our results, we have reported the resolution of EGFP to be 107 ± 17 nm at the same depleting power conditions in absence of gating [18]. 

**Figure 9:**
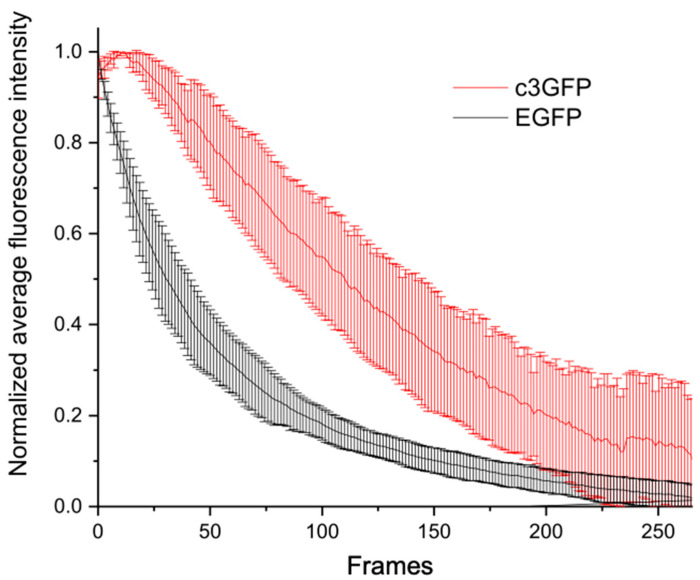
Resistance to STED photobleaching of c3GFP and EGFP. Temporal profile of fluorescence of Vim-c3GFP (red) and Vim-EGFP (black) upon repeated STED acquisitions. Each point represents the average of five STED images and its standard deviation is shown as error bar.

Finally, we compared the resistance of c3GFP and EGFP fused to vimentin to bleaching induced by STED (Figure 9). More specifically, we repeated acquisitions (frames) of the same cellular field, adopting the same conditions of previous STED images except for the number of averages, which was set to 1. Remarkably, the fluorescence of Vim-EGFP was found to drop quickly, becoming almost negligible (<5%) after about 200 acquisitions (Figure 9, black line). In stark contrast, the fluorescence of Vim-c3GFP decreased much more gently after a minor transient increase of about 10–15 acquisitions (Figure 9, red line). After 96 frames, which is the typical number of acquisitions we averaged for each cell image, c3GFP and EGFP retained about 55% and 18% of the original fluorescence, respectively. This implies that c3GFP is at least three-fold more stable than EGFP under optimized STED imaging conditions.

## 3. Discussion

In the last thirty years, two scientific revolutions have changed forever the way we look and study biological matter. The seminal work on the Green Fluorescent Protein (GFP) by of Osamo Shimomura, Martin Chalfie, and Roger Tsien (recipients of the Nobel prize in Chemistry in 2008) enabled, for the first time, selective labeling of target proteins and the ability to follow their biochemical pathways in real time, on account of the genetically encodable property of GFP. In a few years, engineering of the original GFP sequence, as well as of those of other fluorescent proteins coming from other sea organisms, afforded a mesmerizing toolbox of fluorescent reporters (FPs), which were (and are) tailored to a wide range of functional imaging applications. At approximately the same time, the development of imaging techniques able to break the diffraction barrier of light (super-resolution imaging or optical nanoscopy) enabled fluorescent microscopy to investigate biological features with unprecedented details. It comes as no surprise that in recent years, many scientists tried to combine the genetic encoding of FPs with the photophysical properties of the fluorescent reporter required by super-resolution techniques. This challenge proved successful for several super-resolution approaches, such as PALM, SIM, and RESOLFT. Yet, the peculiar characteristics of STED imaging, namely the use of a very intense depletion beam and the need to gate photon arrival to maximize contrast, were found to comply only partially with the photophysical properties of most FPs. This is in stark contrast to the generality of the depletion mechanism by stimulated emission, which in principle makes straightforward the technical implementation of STED imaging in conventional confocal microscopes. Thus, the development of FP variants able to withstand STED imaging is a relevant goal in the field of fluorescent protein engineering.

The mutant F99S/M153T/V163A of the GFP, referred to as c3GFP, developed early from its ancestor GFP by DNA shuffling [45]. Expression in cells showed that c3GFP is about 42 times more fluorescent than GFP [45]. The X-ray structure of c3GFP was found to be very similar to GFP, because the three mutated residues lie on the protein’s surface and the chromophore position is nearly unaffected by their presence [46]. In keeping with the structural similarity to GFP, c3GFP is characterized by the concomitant, pH-independent presence of neutral and anionic chromophore, as highlighted by two bands in the visible region of the absorption spectrum (Figure 1a). Our 2S model of chromophore protonation attributes this co-presence to the buffering action of the lateral ionizable chain of a nearby amino acid, most likely E222 [25]. wtGFP and c3GFP are similar also for the excited state photophysics. In both proteins, excitation of the neutral **A** state triggers ESPT process, yielding an excited transient form I* whose electronic properties are typical of the anionic chromophore in state **B**, but the chromophore environment resembles that of the neutral state **A** (Scheme 1). As a result, excitation of both **A** and **B** yields green fluorescence with nearly superimposable emission spectra (Figure 1b). Yet, our fast spectroscopy analysis of c3GFP clearly revealed the absence of excited state absorption below 600 nm, a valuable feature in view of STED imaging systems dedicated to green fluorophores and endowed with <600 nm depletion laser. Additionally, we found out the fluorescence lifetimes of **A** and **B** states to nearly coincide at about 3.3 ns.

CW g-STED experiments in vitro on c3GFP alone, and in cellulo on a fusion chimera of Vimentin with c3GFP (Vim-c3GFP), experimentally confirmed our intuition of c3GFP as an efficient probe for STED imaging. We found out that c3GFP outperformed the standard probe EGFP in terms of resolution at the same depletion intensity and photostability by excitation at 488 nm. Furthermore, the longer lifetime of c3GFP allows for larger gate times (up to 2 ns) in g-STED, affording higher contrast, lower depletion intensity, and ultimately better resolution than EGFP. Of note, the lifetime of c3GFP is comparable with the longest-decaying members of the *Aequorea* family, mostly yellow mutants, such as EYFP (τ = 3.27 ns, [47]), Citrine (τ = 3.61 ns, [22]), E^2^GFP (τ = 3.49 ns, [48]), and wQ (τ = 3 ns, [49]). YFPs are characterized by complex photophysics at excited state, leading to long-lived dark states [22,23] and lower photochemical stability [24], which make them much less suitable for STED imaging. Thus, although a long probe lifetime is in principle always beneficial for g-STED, its actual embodiment in photostable fluorescent proteins posits a challenging criterium for the development of future FP variants. Indeed, complex photophysical processes associated to poor quantum yields may be revealed even in protein mutants whose long lifetime witnesses very efficient radiative pathways (e.g., Citrine, Φ= 0.76), owing to the strong illumination intensity at the focus of the microscope.

## 4. Materials and Methods

### 4.1. Solvents

All solvents were spectroscopic grade and were purchased from Sigma-Aldrich (Milan, Italy).

### 4.2. Protein Construction and Expression

The recombinant c3GFP was obtained by cloning c3GFP in pET28c between NheI and NotI, taking advantage of the 6Htag at the N-terminal of the vector. Recombinant c3GFP was transformed in E. coli BL21(DE3) strain (Thermo Fisher, Milan, Italy) and the cells were grown at 37 °C to an absorbance at 600 nm of 0.6. Protein expression was induced with 250 µM isopropyl-β-D-thiogalactoside (IPTG, Merck, Milan, Italy) for 16 h at 28 °C. Cells were harvested by centrifugation (4500× *g*., 20 min, 4 °C) and frozen at −20 °C. Cells were then resuspended in ice cold lysis bufferA (50 mM tris-HCl pH 8.0, 150 mM NaCl, EDTA-free protease inhibitor cocktail (Roche Italia, Monza, Italy)) and lysed by sonication on ice followed by 1 h treatment with 0.1% Triton-X100 at 4 °C. After removal of the debris by centrifugation (12,000× *g*, 1 h, 4 °C), the supernatant was mixed with 5 mL of NiNTA Agarose beads (QIAGEN, Milan, Italy) and incubated on a rotor for overnight at 4 °C. The His-tagged protein was then eluted in buffer A + 500 mM Imidazole. The eluted protein was exchanged in 20 mM diethanolamine (DEA), pH 8.5. Protein concentration was determined by UV absorption measurements.

### 4.3. Protein for Eukaryotic Expression

EGFP-Vimentin-7 was a gift from Michael Davidson (Addgene plasmid # 56439; http://n2t.net/addgene:56439; RRID:Addgene_56439; accessed on 1 February 2022). The Vimentin-7-c3GFP construct was generated by replace EGFP with c3GFP. c3GFP was polymerase chain reaction (PCR) amplified to introduce *AgeI/NotI* restriction sites to the 5′ and 3′ ends, respectively. These unique restriction sites were present also in the 5′ and 3′ ends of EGFP in Vimentin-7-EGFP. All PCR primers were purchased from Merck (Milan, Italy) and all restriction endonucleases from New England Biolabs (Euroclone, Milan, Italy).

### 4.4. Cell Cultures and Transfections

Lung adenocarcinoma human cells A549 and H1975 were obtained from American Type Culture Collection (ATCC, Manassas, VA, USA) and grown in Roswell Park Memorial Institute (RPMI) 1640 medium (RPMI 1640, Invitrogen, Carlsbad, CA, USA), supplemented with 10% Fetal Bovine Serum (FBS), glutamine (2 mM), 100 U/mL penicillin, and 100 mg/mL streptomycin (Thermo Fisher, Milan, Italy) at 37 °C in a humidified atmosphere with 5% CO_2_; the medium was renewed three times per week. 

### 4.5. Cell Transfections

A total of 12 × 10^4^ cells were plated 24 h before transfection onto a 35-mm glass-bottom dish (WillCo-dish GWSt-3522, WillCo Wells B.V., Amsterdam, The Nethelands). Transfections of all constructs were carried out using Lipofectamine reagent (Thermo Fisher, Milan, Italy) according to the manufacturer’s instructions. After 24 h from transfection, cells were fixed by PFA 2% in PBS 1× and imaged or imaged without fixation.

### 4.6. Steady-State Absorption and Fluorescence Spectra

Absorption and fluorescence spectra were recorded in cuvettes with 1 cm optical path (Hellma, Müllheim, Germany). Absorption was collected at 25 °C on a JASCO V550 spectrophotometer (JASCO Europe, Cremella (LC), Italy) using 1 nm band-pass and 0.25 s integration time. Fluorescence intensity spectra were recorded at 25 °C by a Fluoromax-4 fluorometer (Jobin-Yvon, Milan, Italy) with 1–2 nm excitation/emission bandpass and 0.2–0.5 s integration time. In a typical spectroscopic experiment, 1–5 µL of mother protein solution was dissolved in 1.2 mL of buffer at desired pH and placed in a 1-cm quartz cuvette of 1.5 mL volume (Hellma, Milan, Italy) to obtain maximum absorbance values 0.1–0.2 in the visible range of spectrum. High pH buffer (pH 8) was 20 mM diethanolamine (DEA). Low pH buffer (pH 5.2) was 4 mM citrate-20 mM phosphate buffer. In both cases, pH was adjusted to the desired value by small aliquots of sulfuric acid or NaOH 1 N.

### 4.7. Molar Absorption Coefficients and Quantum Yields

Molar absorption coefficients were determined from sample absorbances by using the actual concentration of the protein quantified by the denaturation method of Ward [32]. Briefly, all GFPs in NaOH 0.2 N display a single visible absorption peak at 447–448 nm with a 44,100 M^−1^ cm^−1^ extinction coefficient. Actually, this method directly measures chromophore concentration, which is assumed to correspond to the amount of *folded* protein. Protein quantum yields were determined by using Fluorescein as standard (Fluo = 0.92 in NaOH 0.1 M). More in detail, the absorption and fluorescence emission spectra of a protein solution (at a given pH) and fluorescein (in NaOH 0.1 M) were collected sequentially; the absorption of both samples was kept below 0.08 to avoid inner filter effects. The protein quantum yield was calculated by the equation:

$$\Phi_{P}=\Phi_{Fluo}\frac{F_{P}}{A_{P}}\frac{A_{Fluo}}{F_{Fluo}}$$

where *F_P_* and *F_Fluo_* are the integrated fluorescence intensities from 465 to 700 nm, *A_P_* and *A_Flu_* are the absorbances at the excitation wavelength, and the pedices *Pi* and *Fluo* refer to the fluorescent protein and fluorescein, respectively.

### 4.8. Fluorescence Lifetime Detection

Fluorescence lifetime has been recorded using a digital frequency domain acquisition module (AlbaFLIM, ISS Inc., Champaign, IL, USA), coupled to a NikonA1r MP confocal-2p microscope (Nikon Corporation, Tokyo, Japan). For excitation, we used a 405 nm (LDH-P-C-405M, PicoQuant GmbH Berlin, Germany) and a 488 nm (ISS Inc., Champaign, IL, USA) pulsed lasers running at 40 MHz. Phasor analysis and multifrequency lifetime fit was performed with VistaVision software (ISS Inc., Champaign, IL, USA).

### 4.9. Ultrafast Spectroscopy

The experimental setup for the femtosecond transient absorption and fluorescence up-conversion measurements has been described elsewhere [50,51]. In particular, a 800-nm radiation is amplified by the Ti:Sapphire laser system (Spectra Physics) and then converted into 400-nm excitation pulses (ca. 60 fs) by Apollo (2nd and 3rd Harmonic generator). A small portion of the fundamental laser beam (800 nm light) enters the transient absorption spectrometer (Helios, Ultrafast Systems, Sarasota, FL, USA), passes through an optical delay line (time window of 3200 ps), and is finally focused onto a Sapphire crystal (2 mm thick) to generate a white-light continuum (450–800 nm), used as the probe. The temporal resolution is about 150 fs and the spectral resolution 1.5 nm. In the fluorescence up-conversion setup (Halcyone, Ultrafast Systems, Sarasota, FL, USA), the 400-nm pulse excites the sample whereas the fundamental laser beam acts as the “gate” light, after passing through a delay line, which is then summed to the sample emission promoting the up-conversion process. The time resolution is about 200 fs, while the spectral resolution is 1.5 nm. All measurements were carried out under the magic angle condition in a 2-mm cell considering OD~0.6 and λ_pump_ = 400 nm The solution was stirred during the experiments to avoid photoproduct interferences. Photodegradation was checked recording the absorption spectra before and after the time-resolved measurement, where no significant change was observed. The experimental 3D data matrices were analyzed performing Global Analysis through the Surface Xplorer PRO (Ultrafast Systems, Sarasota, FL, USA) software to obtain the Decay Associated Spectra (DAS) and performing Target Analysis through the GloTarAn software to obtain the Species Associated Spectra (SAS).

### 4.10. g-STED Measurements

Measurements were performed by means of a Leica TCS SP5 STED (Leica-microsystems, Mannheim, Germany) inverted confocal/STED microscope. Samples were viewed with a 100× Apochromat NA = 1.4 oil-immersion objective. Excitation was provided by a White Light Laser and selecting the 488 nm wavelength by an acoustic-optical tunable filter (AOTF). Detection was done in the 500–550 nm range by one avalanche photodiode detector. Pinhole was set to 0.6–1 Airy size. Line scanning speed ranged from 100 to 1400 Hz in standard acquisition mode. In g-STED mode, the 592 nm CW laser beam was superimposed at a typical power of 180–190 mW before the objective. Detector gating time was set to 1 ns for the comparison EGFP/c3GFP or to 2 ns for c3GFP imaging. Image analysis was carried out by the ImageJ free software (NIH, Bethesda, MD, USA). Fourier Ring Correlation was carried out by the FRC_plugin of ImageJ available from the PTBIOP Update Site (https://www.epfl.ch/research/facilities/ptbiop/, accessed on 1 February 2022).

## 5. Conclusions

We here demonstrated that c3GFP, a rather neglected variant of GFP, represents a valuable alternative to more common EGFP and YFP/Citrine mutants for STED imaging studies targeting the green-yellow region of the optical spectrum. Indeed, c3GFP displays strong photostability upon <600 nm depletion on account of no excited state absorption at those wavelengths, as well as long lifetime of fluorescence emission, which enables gated STED imaging at lower depletion intensities. Accordingly, c3GFP adds a further important imaging option to the present toolbox of fluorescent proteins for in cellulo and in vivo biological studies.

## Data Availability

Data contained within the article and supplementary materials are available on request from the authors.

## Appendix A. Theoretical Description of In Vitro Depletion Curve

We shall consider an excited fluorophore with original lifetime τ which is exposed to a depletion beam of power I. The consequence of the depletion beam is the quenching of fluorescence, which can be represented by a shortened lifetime $\tau_{D}$ expressed by:

(A1) $$\frac{1}{\tau_{D}}=\frac{1}{\tau }+\sigma \frac{I}{hc}$$

where σ is the depletion cross-section. This leads to a quenching factor of:

(A2) $$\frac{F_{D}}{F}=\frac{\tau_{D}}{\tau }=\frac{1}{\sigma \tau \frac{I}{hc}+1}$$

where $F_{D}$ and F are the depleted and non-depleted emissions, respectively. By imposing $F_{D}/F=0.5$, we recover the canonical STED relation that defines the saturation intensity, i.e., $I=I_{sat}=hc/\sigma \tau$.

If now we gate the arrival time of photons from $t=\tau_{g}$ to t=∞, the fraction of collected fluorescence will depend on the gate time according to:

(A3) $$F_{D}=A\int_{\tau_{g}}^{+\infty }exp\left(-\frac{t}{\tau_{D}}\right)=A\tau_{D}\cdot exp\left(-\frac{\tau_{g}}{\tau_{D}}\right)$$

(A4) $$F=A\int_{\tau_{g}}^{+\infty }exp\left(-\frac{t}{\tau }\right)=A\tau \cdot exp\left(-\frac{\tau_{g}}{\tau }\right)$$

where A is a preexponential factor that depends only on excitation and not on the depletion intensity.

Accordingly, we have:

(A5) $$\frac{F_{D}}{F}=\frac{\tau_{D}}{\tau }\cdot exp\left[-\tau_{g}\left(\frac{1}{\tau_{D}}-\frac{1}{\tau }\right)\right]$$

which, remembering the definition of $\tau_{D}$ given in (A1), becomes:

(A6) $$\frac{F_{D}}{F}=\frac{\tau_{D}}{\tau }\cdot exp\left[-\frac{\sigma \tau_{g}}{hc}I\right]$$

Thus, the original quenching factor at $\tau_{g}=0$ is further scaled by:

(A7) $$exp\left[-\frac{\sigma \tau_{g}}{hc}I\right]$$

Accordingly, $I_{sat}\left(\tau_{g}\right)<I_{sat}\left(\tau_{g}=0\right)$.
